# Bispecific Anti-HIV Immunoadhesins That Bind Gp120 and Gp41 Have Broad and Potent HIV-Neutralizing Activity

**DOI:** 10.3390/vaccines9070774

**Published:** 2021-07-12

**Authors:** Seth H. Pincus, Ryan B. Craig, Lauren Weachter, Celia C. LaBranche, Rafiq Nabi, Connie Watt, Mark Raymond, Tami Peters, Kejing Song, Grace A. Maresh, David C. Montefiori, Pamela A. Kozlowski

**Affiliations:** 1 Department of Chemistry and Biochemistry, Montana State University, Bozeman, MT 59715, USA; cmwatt29@gmail.com (C.W.); markcraymond@gmail.com (M.R.); tami.peters@montana.edu (T.P.); 2 Research Institute for Children, Children’s Hospital, New Orleans, LA 70118, USA; rycraig@gmail.com (R.B.C.); lweachter@gmail.com (L.W.); kjingsong@gmail.com (K.S.); gmaresh10@gmail.com (G.A.M.); 3 Department of Pathology, Tulane University, New Orleans, LA 70112, USA; 4 Department of Surgery, Duke University, Durham, NC 27707, USA; celia.labranche@duke.edu (C.C.L.); david.montefiori@duke.edu (D.C.M.); 5 Department of Microbiology, Immunology, and Parasitology, LSU School of Medicine, New Orleans, LA 70112, USA; rnabi1@lsu.edu (R.N.); pkozlo@lsuhsc.edu (P.A.K.)

**Keywords:** HIV, immunotherapy, immunoadhesin, AIDS

## Abstract

We have constructed bispecific immunoglobulin-like immunoadhesins that bind to both the HIV-envelope glycoproteins: gp120 and gp41. These immunoadhesins have N terminal domains of human CD4 engrafted onto the N-terminus of the heavy chain of human anti-gp41 mAb 7B2. Binding of these constructs to recombinant Env and their antiviral activities were compared to that of the parental mAbs and CD4, as well as to control mAbs. The CD4/7B2 constructs bind to both gp41 and gp140, as well as to native Env expressed on the surface of infected cells. These constructs deliver cytotoxic immunoconjugates to HIV-infected cells, but not as well as a mixture of 7B2 and sCD4, and opsonize for antibody-mediated phagocytosis. Most surprisingly, given that 7B2 neutralizes weakly, if at all, is that the chimeric CD4/7B2 immunoadhesins exhibit broad and potent neutralization of HIV, comparable to that of well-known neutralizing mAbs. These data add to the growing evidence that enhanced neutralizing activity can be obtained with bifunctional mAbs/immunoadhesins. The enhanced neutralization activity of the CD4/7B2 chimeras may result from cross-linking of the two Env subunits with subsequent inhibition of the pre-fusion conformational events that are necessary for entry.

## 1. Introduction

The HIV envelope (Env) glycoproteins, precursor (gp160), surface (gp120), and transmembrane (gp41), are the sole virus-encoded proteins expressed on the surface of virions and HIV-infected cells. Env is the only viral target of HIV-neutralizing antibodies (Abs). As such, the anti-Env Ab response is the subject of intense investigation, both for the development of an HIV vaccine [1,2,3,4,5,6] and for the administration of therapeutic Abs [2,3,7,8,9,10,11,12,13,14,15]. Well-defined regions of Env have been described as targets of patient-derived neutralizing monoclonal Abs (mAbs), including the gp120 CD4 binding site, variable loops, glycan shield, interface of gp120/gp41, and the gp41 membrane proximal external region. Preclinical and clinical trials of mAb therapies for HIV have demonstrated that a single mAb cannot reliably control viremia, whereas combinations of mAbs are more likely to afford prolonged viral suppression [6,7,9,11,16,17,18,19,20]. To address this, bispecific (or greater) anti-envelope Abs/immunoadhesins, consisting of two (or more) domains that bind different portions of gp160, have been created, and some demonstrate broad and potent neutralization [8,10,21,22,23,24,25,26,27,28,29,30,31,32]. Natural mAbs and bispecific constructs that bind to both gp120 and gp41 have been described [24,25,33,34,35,36]. Some of these neutralize quite well [25,34,35], others only poorly [24,33].

In addition to neutralization of virus infectivity, Abs may mediate anti-HIV effects through other mechanisms, particularly those that lead to the elimination of HIV-infected cells. This may be accomplished via Fc-mediated mechanisms, including the induction of complement and antiviral effects of FcR-expressing cells [16,37,38,39,40,41,42,43,44,45], or by the delivery of cytotoxic agents to HIV positive cells. In the course of developing anti-HIV immunoconjugates that use anti-Env mAbs to deliver cytotoxic agents to infected cells (cytotoxic immunoconjugates), we have noted that mAbs to the gp41 immunodominant loop region are most effective, particularly when used in association with soluble CD4 [46,47,48,49]. In an effort to design a better Ab for delivering immunoconjugates, we made a series of genetic constructs in which NH_2_-terminal domains of human CD4 were joined to the NH_2_-terminus of the heavy or light chains of the anti-gp41 mAb 7B2. These bispecific immunoadhesins bind to both gp120 and gp41. Surprisingly the neutralizing abilities of the CD4/7B2 constructs were both broad and potent, far more so than CD4 itself, even though the parental mAb 7B2 has little if any neutralizing activity. As opposed to neutralization, the ability of the bispecific immunoadhesin to deliver a cytotoxic immunoconjugate was diminished when compared to the free components. These constructs may have therapeutic utility for the prevention of HIV infection, treating active infection by neutralizing virus entry into uninfected cells, and targeting infected cells for elimination. The data could suggest that mAbs may neutralize HIV infectivity by limiting the gp120/gp41 structural changes and dissociation that occur upon receptor binding and are necessary for virus infection.

## 2. Materials and Methods

### 2.1. Cells and Reagents

HEK/293T cells were a gift from James Robinson (Tulane University) and were maintained in DMEM, 10% fetal calf serum (GIBCO) in 5% CO_2_ at 37 °C for transient transfection. H9/NL4-3 cells were persistently infected with the NL4-3 molecular clone of HIV [50] and retained a productive infection in virtually 100% of tissue culture cells [46,51]. 293/92UG cells were the kind gift of Bing Chen and constitutively express the clade A Env 92UG in its native conformation at the cell surface [52]. THP-1 cells were obtained from ATCC. Cells were maintained at 37 °C in 5% CO_2_ as described [24,46].

Soluble, two-domain CD4_183_ [sCD4, NIH-ARP, [53]] and CD4-IgG2 [PRO542, Progenics Pharmaceuticals, Tarrytown, NY, USA, [54,55,56]] were used to observe CD4-mediated effects. CH01-31 is an equal concentration mixture of human anti-gp160 mAbs CH01 and CH31 [57]. 8ANC195 was a gift from P. Bjorkman and binds to determinants at the gp120/gp41 interface [36]. Chimeric RAC18 (chRAC18, an anti-ricin A chain murine Ab chimerized to human IgG1/κ) and 23.1D (anti-Lassa) were used as isotype controls [58,59]. Goat anti-human IgG (heavy + light chains) Ab was conjugated to fluorescein isothiocyanate (FITC; Invitrogen) or alkaline phosphatase (AP; Invitrogen). Deglycosylated ricin A chain (RAC) was conjugated to purified anti-human IgG (Invitrogen) for indirect cytotoxicity assays, as described [24,46].

### 2.2. Design and Production of Immunoadhesins

**Figure 1:**
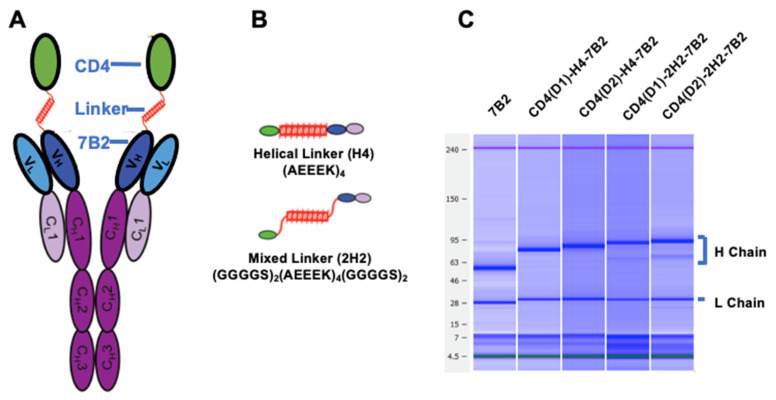
Construction of Ig-adhesins (**A**). Schematic diagram of CD4/7B2 chimeric adhesin, showing CD4 linked to the H chain of anti-gp41 mAb 7B2 (**B**). Schematic diagram of linkers successfully used to join CD4 to 7B2 variable regions (**C**). Microcapillary electrophoresis demonstrating the molecular weights of the heavy and light chains under reducing conditions.

Design and production of CD4/7B2 immunoadhesins were as previously described for double variable domain Abs [24]. Figure 1 and Table 1 summarize the design and nomenclature of the different immunoadhesins. Parental sequences were derived from domain 1 and 2 of human CD4 (Genbank accession number BC025782) or from the modified CD4 designated CD4(D1.22) [60], and from the IgG1/kappa human Ab 7B2 (Genbank accession numbers JX188438 and JX188439), which binds gp41 immunodominant region at AA 598-604 (CSGKLIC) [49]. CD4 constructs were joined to 7B2 using linkers described previously [24]. The first linker is flexible with little structure: (GGGGS)_4_, and the second consists of a helical core surrounded by two sets of flexible regions, and is designated as 2H2: [(GGGGS)_2_-A(EAAK)_4_A-(GGGGS)_2_]. The final linker is the helical core only and designated H4: [A(EAAK)_4_A]. The CD4 domains were joined to the N-terminus of either the 7B2 IgG1 heavy chain, 7B2 kappa light chain, or both, creating a set of chimeric CD4/7B2-Igs. Only constructs in which CD4 was linked to the heavy chain and using linkers containing a helical region were produced in sufficient quantity for further study. Table 1 provides details of the chimeric proteins used in the studies. Two additional mutations (T250Q and M428L) were introduced into the constant region of the heavy chain to increase in vivo half-life of Ab [61]. Protein sequences were designed in silico and DNA synthesized de novo (GenScript, Piscataway, NJ, USA). DNA sequences were codon-optimized and cloned into the eukaryotic expression plasmid pcDNA3.1 (Invitrogen, Waltham, MA, USA). Heavy and light chain plasmids (30 µg each) were mixed in 4-mL serum-free DMEM with 200 µg polyethylenimine (PEImax, Polysciences Inc., Warrington, PA, USA), incubated for 15 min at room temperature with occasional swirling, and added to 293T cells at 70% confluence in TC150 flasks in 5% protein-G adsorbed fetal calf serum. Supernatant was collected at days 3 and 7 and purified by affinity chromatography on Protein G agarose. Ab concentrations were measured by bicinchoninic acid protein assay (Pierce, Rockford, IL, USA), confirmed using OD_280_, and by microcapillary electrophoresis (Agilent Bioanalyzer, GE Healthcare, Chicago, IL, USA), which also confirmed molecular size and purity, Figure 1C.

### 2.3. ELISA

Abs and adhesins were tested by indirect ELISA for binding to the gp41 peptide representing the epitope bound by mAb 7B2 or to trimeric gp140. The gp41 peptide is a linear sequence [LGIWGCSGKLICTT]. Gp140 is trimeric, folded SF162 [NIH-ARP and a gift from Dr. L. Stamatatos, [62]]. Immulon 2HB plates (Thermo, Waltham, MA, USA) were coated with 0.1 µg/100 µL of peptide or 0.05 µg/100 µL of recombinant antigen, and the assay performed as described elsewhere [24]. The binding of immunoadhesins or mAbs was detected using AP-conjugated goat anti-human IgG (H + L chain specific) secondary Ab and 0.5 mg/mL of chromogenic substrate p-nitrophenyl phosphate in succession. ELISA plates were read at 405 nm at room temperature in a BioTek EL320 microplate reader (BioTek, Winooski, VT, USA). Data are presented as the mean and SEM of triplicate assays.

### 2.4. Indirect Immunofluorescence and Flow Cytometry

H9/NL4-3, HEK293T, and 293/92UG cells (1 × 10^5^) were stained for flow cytometry in 200 µL round bottom 96-well plates (Costar, Lowell, MA, USA). Serial dilutions of test reagent in PBS, 1% bovine serum albumin, 0.01% Na azide were added to the cells. Cells were incubated for 2 h at room temperature, washed, stained with FITC-conjugated goat anti-human IgG (H + L chain specific) secondary Ab for 2 h, and then washed twice and fixed in 100 µL of 2% paraformaldehyde (PFA). Cells were analyzed on a Becton-Dickinson LSR II. A total of 10,000 events was collected and the data analyzed using FloJo software (Treestar, Ashland, OR, USA). Forward scatter (FSC) and side scatter (SSC) gated data are presented as histograms or graphs of mean fluorescence intensity.

### 2.5. Cytotoxicity Assay

An indirect cytotoxicity assay was performed to screen unconjugated Abs for their ability to kill infected cells [24,46,63]. Cells (8–12 × 10^3^ per well depending upon cell type) were plated in triplicate in 96-well flat bottom plates. Controls included: no cells (background) and cells in the absence of Ab (uninhibited). Abs (200 ng/mL) were incubated with cells for 1 h in RPMI at 37 °C. The cytotoxic immunoconjugate was affinity purified goat anti-human IgG (Invitrogen) conjugated to deglycosylated ricin A chain using the long chain heterobifunctional cross linking reagent, succinimidyl 6-[3(2-pyridyldithio)proprionamido]hexanoate (Pierce). The cytotoxic secondary conjugate was added to a final concentration of 300 ng/mL. The plates were then incubated for 3 days. For the final 6 h of incubation, MTS/PMS substrate (Promega, Madison, WI, USA) was added to each well and plates read at 490 nm. Results represent the mean and SEM of triplicate samples.

### 2.6. Neutralization Assays

Neutralization of Env-pseudo-typed reference viruses in TZM-bl cells was performed at the Duke University HIV neutralization reference laboratory, using established validated assays and standardized pseudovirus [64,65]. Results are reported as the IC_50_, the lowest concentration yielding >50% inhibition of infectivity. Comparison of data to historical controls was performed using CATNAP [66]. Statistical comparison of breadth versus potency curves was performed using a two-tailed, non-parametric Wilcoxon signed rank test with pairs matched for each isolate.

### 2.7. Ab-Dependent Phagocytosis

Assays for Ab-dependent phagocytosis (ADP) were performed using the THP-1 monocyte cell line and HIV gp140-coated fluorescent beads using methods similar to those described [67]. Briefly, 1.8 × 10^8^ neutravidin-coated 1 µm Fluorospheres (Invitrogen) were treated with 3.5 µg biotin-conjugated rabbit anti-His tag Ab (Pierce), then washed and reacted with 7 µg His tagged-recombinant gp140_SF162_ protein (Immune Technology, New York). After washing, 10^6^ beads per well were incubated at 37 °C for 1 h with triplicate dilutions of Ab in a V-bottom plate. The Abs were diluted in RPMI 1640 medium containing 10% Ultra-Low (IgG-depleted) FBS (Gibco), penicillin, streptomycin, and L-glutamine. THP-1 cells (2 × 10^4^ per well) were then added in the same medium and incubated at 37 °C in 5% CO_2_. After 6 h, the cells were washed with Ca^+2^/Mg^+2^-free DPBS and incubated at 37 °C for 10 min with 50 µL of 0.05% Trypsin/EDTA (Gibco). Cells were washed twice in DPBS, resuspended in 1% PFA, then examined by flow cytometry. The phagocytic score was calculated by multiplying the number of bead-positive cells by the median fluorescent intensity. The phagocytic score of each sample was then divided by the average phagocytic score obtained with THP-1 in medium alone to obtain the phagocytic score ratio.

## 3. Results

### 3.1. Production and Characterization of Immunoadhesins

We have previously described a panel of double variable domain (DVD) Abs that bind to both gp120 and gp41 using variable domains derived from mAbs to the CD4-binding site of gp120 (mAb b12) and to the gp41 external loop region (mAb 7B2) [24]. Although these constructs bound both gp120 and gp41, we did not improve their ability to neutralize HIV nor to deliver cytotoxic agents to infected cells. However, we did demonstrate that the DVD’s worked most efficiently when the gp120-binding domain was at the NH_2_-terminus, joined to the anti-gp41 domain using linkers containing helical, rather than flexible, peptide domains. Consequently, we designed new constructs to improve neutralization and effector functions incorporating these design principles, but containing domains of human CD4, rather than anti-gp120 mAb joined to 7B2 (Figure 1).

We used two forms of CD4: native and CD4(D1.22), which has improved affinity and neutralizing activity [60]. CD4 was joined to the amino terminus of either H, L, or both chains of full-length mAb 7B2, a human IgG1/κ anti-gp41 mAb. The H and L chains were expressed from different plasmids by cotransfection into 293T cells, thus creating CD4/7B2 chimeric Ig-like immunoadhesins with various CD4-Ab conformations and linkers joining CD4 to 7B2. We tested three different linkers: a flexible linker (GGGGS)4, a helix-creating sequence [A-(EAAAK)_4_-A], and a combination of helical and flexible sequences [(GGGGS)_2_-(EAAAK)_4_-(GGGGS)_2_]. We only obtained the secretion of CD4/7B2 chimeras when CD4 was attached to the heavy chain and when the linker contained helical domains. Constructs with CD4 attached to either the light chain or to both heavy and light chains failed to produce antigen-binding protein, as did linkers that solely consisted of flexible domains. Table 1 describes the nomenclature and construction of the chimeras.

Adhesins produced in cell supernatants were purified on protein G agarose and concentrated. Microcapillary electrophoresis confirmed molecular size and purity of the H and L chains of the novel constructs (Figure 1C). To compare the functional activity of the four chimeras described in Table 1 to the parental antibodies, the following samples were tested side-by-side with them: 1. Irrelevant isotype matched Ig; 2. mAb 7B2 alone; 3. 7B2 +sCD4_183_; 4. CD4-IgG2; and 5. 8ANC195, a human mAb that binds at the gp120/gp41 interface [36]. Because the activity assays utilize secondary antibodies, we have kept the concentration of Fc in each preparation the same. In the figures that follow, immunoadhesins are shown with solid lines and filled symbols, and controls with dotted lines/open symbols.

### 3.2. Binding of Immunoadhesins to Antigen

**Figure 2:**
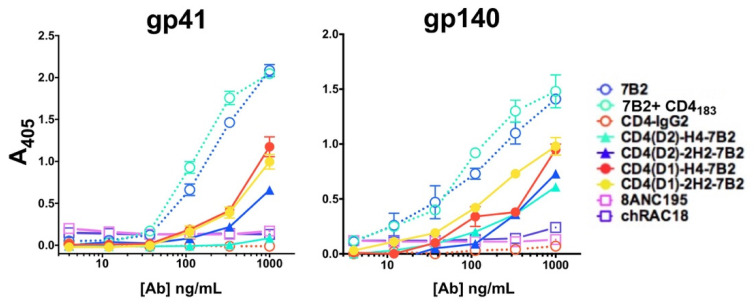
Binding of Abs and Ig-adhesins to envelope antigens by ELISA. We evaluated the binding of a panel of anti-Env mAbs and Ig-adhesins to either a peptide representing the gp41 epitope of 7B2 or trimeric gp140. The novel Ig-adhesins are represented as solid lines and filled symbols, others as dotted lines and open symbols. Binding to the antigen is measured as A_405_, shown as the mean and SEM of triplicate samples. The results shown here are representative of >5 independent experiments.

ELISA was used to demonstrate binding of each construct to the gp41 peptide, which is the target of 7B2, or to trimeric quasi-native gp140. ELISA plates were coated with respective antigens, incubated with serial dilutions of Abs, and probed with an AP-conjugated anti-human IgG secondary Ab (Figure 2). ELISA binding primarily reflected the binding activity of 7B2. When tested on the gp41 peptide representing the 7B2 epitope, the mAb bound far better than the immunoadhesins, and one (CD4(D2)-H4-7B2) failed to bind at all, indicating that the 7B2 binding site is partially occluded by CD4. As expected, neither CD4-IgG2 nor 8ANC195 bound the peptide. Binding to gp140 was similarly limited, with 7B2 mAb producing the best binding, and the adhesins less. Because neither CD4-IgG2 nor 8ANC195 bound to the gp140, there has been some loss of conformational integrity of this recombinant antigen.

**Figure 3:**
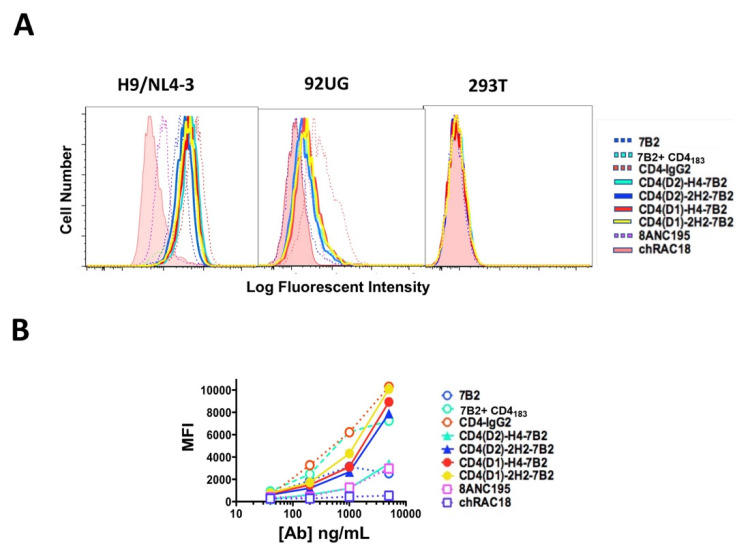
Binding of Abs and Ig-adhesins to Env-expressing cells. Binding of the panel was detected by secondary immunofluorescence and flow cytometry. For each sample, 10,000 H9/NL4-3, 293/92UG, or 293T cells were evaluated (**A**). Histograms of analyses performed with 1 µg/mL of mAb or Ig-adhesin. Cells were first gated based on FSC and SSC, before analyzing for fluorescence. Binding of the isotype control is shown as the solid pink histogram (**B**). Titration curves of binding to NL4-3. For each concentration, the mean fluorescent intensity and SEM are shown. When no error bars are visible, they are smaller than the symbol. The data shown are representative of >5 unique experiments.

To test recognition of native Env by the chimeras, we used indirect immunofluorescence and flow cytometry to analyze binding to persistently-infected H9/NL4-3 cells, constitutively expressing Env-transfectants 293/92UG and parental HEK293T cells (Figure 3). No antibodies or immunoadhesins bound to the uninfected parental cells. The infected H9/NL4-3 expressed higher levels of antigen on the cell surface than do the transfected 293/92UG. CD4-IgG2 bound best to both the infected and transfected cells, followed closely by 7B2 in the presence of sCD4_183_. MAb 7B2 alone bound only weakly. The four chimeric adhesins bound as well as 7B2 + sCD4_183_, indicating that there is cooperativity among the binding sites on the adhesins.

### 3.3. Immunoconjugate Cytotoxicity

**Figure 4:**
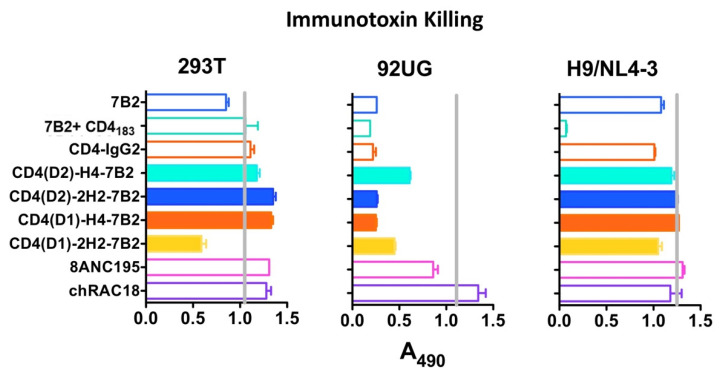
Efficiency of mAbs and Ig-adhesins in targeting immunotoxin killing. Cytotoxicity was examined on different cell types: control HEK-293T cells not expressing Env, HEK-293 cells transfected to constitutively express 92UG Env (designated 92UG), and persistently infected H9/NL4-3. Cells were incubated with the indicated mAb or Ig-adhesin (200 ng/mL), and a secondary immunotoxin, ricin A chain-conjugated goat anti-human IgG (300 ng/mL). MTS dye reduction was measured at 72 h and results are shown as A_490_, mean, and SEM. Less absorbance indicates more effective killing. The vertical grey line represents MTS dye reduction in the absence of a primary antibody. Results are representative of >5 individual experiments.

We have been developing immunoconjugates to deliver cytotoxic agents to HIV-infected cells as a means to eradicate HIV infection. We have compared many mAbs and have shown that the most potent immunotoxin killing is observed with mAb 7B2 when combined with sCD4 [46,47,48,49,68]. Our rationale for constructing these chimeras was to create a single molecule that could deliver the cytotoxic moiety. To study whether the CD4/7B2 chimeras function in this regard, we used an indirect immunoconjugate assay to compare these constructs. H9/NL4-3, 293T, or 293/92UG cells were incubated with serial dilutions of Ab and then treated with anti-human IgG conjugated to ricin A chain. Cell viability was measured after 3 days (Figure 4). No cytotoxicity was observed with the Env(-) HEK/293T cells. Although it appears that the CD4(D1)-2H2-7B2 construct had modest cytotoxicity against these negative control cells in this experiment, subsequent experiments at concentrations up to 10X that in Figure 4 showed no toxicity. The H9/NL4-3 cells were less susceptible to immunotoxin killing than 293/92UG, despite having higher levels of Env on the cell surface (Figure 3), which is an observation we have previously made [46]. Only 7B2 + sCD4_183_ killed off H9/NL4-3. HEK293/92UG cells were killed by all mAbs except the isotype control and 8ANC195. None of the chimeric adhesins functioned any better than the parental mAbs. These data indicate that our original rationale for creating the chimeras may have been incorrect.

### 3.4. HIV Neutralization

**Figure 5:**
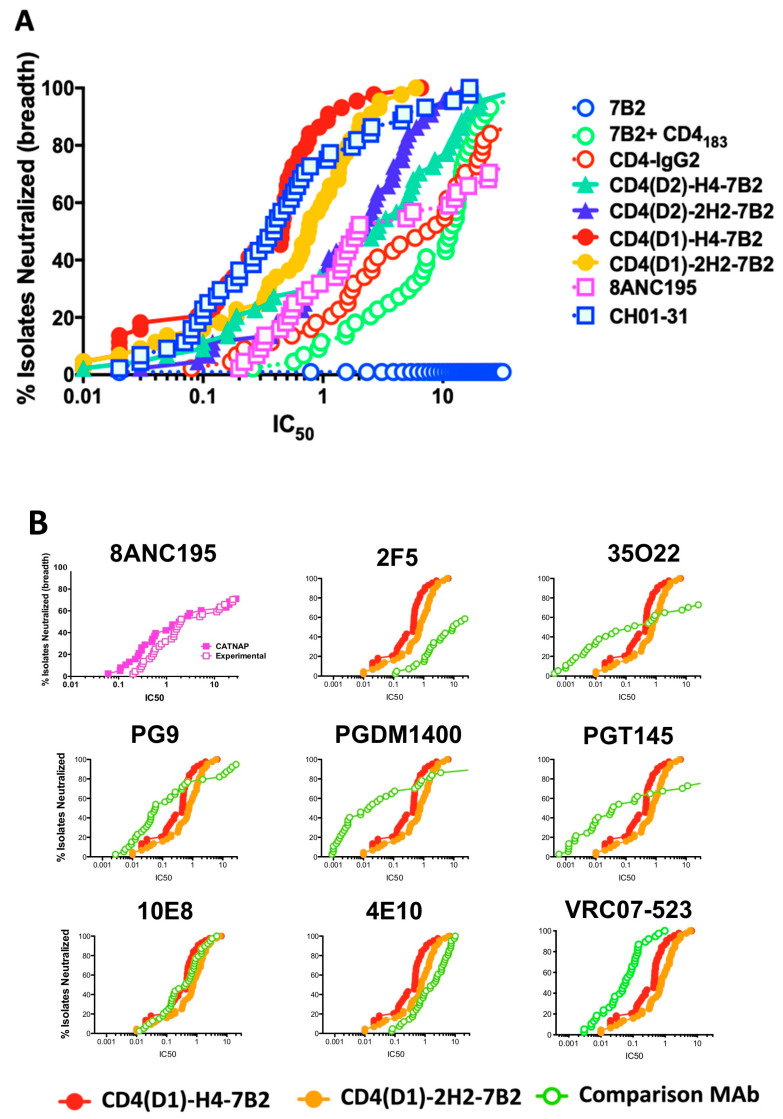
Neutralization by mAbs and Ig-Adhesins. Neutralization was studied using the TZM-bl assay with 44 different tier 1 and 2 pseudotyped HIV isolates (**A**). The panel of mAbs and Ig-adhesins, along with a mixture of CH01+CH31 neutralizing mAbs was tested for potency and breadth of neutralization. Vertical axis is percentage of isolates neutralized, the horizontal is IC_50_ (µg/mL) (**B**). Comparison of experimental results with historical data in CATNAP. The upper left panel compares mAb 8ANC195 experimental vs historical data. The remainder compare two Ig-Adhesins to other potent, broadly-neutralizing mAbs. Axes are the same.

MAb 7B2 neutralizes HIV weakly, if at all, with results dependent upon the assay used. CD4-IgG2 contains both the gp120 binding site and an Ab constant region, and is effective for neutralization. However, CD4-IgG2 is most efficacious against laboratory isolates but had disappointing results in clinical trials [55,56,69]. Thus, there was no reason to expect that CD4/7B2-Ig chimeras would have great neutralizing activity. However, for the sake of completeness, we performed preliminary assays measuring neutralization of live HIV by 7B2, CD4-IgG2, and the immunoadhesins, and observed impressive neutralization results (not shown). We therefore sought to validate these results using a highly reproducible pseudovirus assay with reference strains of pseudovirions so that the neutralizing activity of our immunoadhesins could be compared to that of other broadly-reactive, highly-potent, neutralizing mAbs [64,65]. Figure 5A shows a potency vs breadth plot of the neutralizing activity of the chimeric adhesins and controls against 44 different tier 1 and 2 pseudoviruses. A spreadsheet of the raw IC_50_ data is included as Supplementary Table S1. The “positive control” in this assay is a mixture of two different highly potent neutralizing mAbs CH01 and CH31. Mab 7B2 has no neutralization activity in this assay. CD4-IgG2 shows modest neutralization with 50% of isolates neutralized at ~3 µg/mL. Mixing 7B2 and CD4_183_ was less effective with 50% of isolates neutralized at 10 µg/mL. However when CD4 and 7B2 were combined into a single molecule, synergistic effects were observed, with all four chimeric immunoadhesins showing significantly greater neutralization (by Wilcoxon matched pairs signed rank test, Table S1) than either 7B2+CD4_183_ or CD4-IgG2. Those adhesins based on the CD4(D1.22) construct had 10–30-fold improved neutralization compared to those based on the native CD4 sequence (*p* < 0.001), consistent with previously published results comparing CD4(D1.22) to wild-type CD4 [60]. The role of the linker between CD4 and 7B2 heavy chain were minor. Comparing the CD4(D1) constructs, the H4 linker appeared better than 2H2, but with CD4(D2) chimeras, the reverse was observed. In Figure 5B, we used the CATNAP database [66] to compare breadth and potency of neutralization by the immunoadhesins to historical data from a panel of well-described, highly-potent, broadly-neutralizing anti-HIV mAbs. All were compared on the same pseudovirus isolates. The graph in the upper left shows our experimental data compared to the historical data for mAb 8ANC195, yielding essentially overlapping curves. The data demonstrate that while some of the mAbs were more potent (e.g., 35O22, PGDM1400, and PGT145), they demonstrated less breadth. Only VRC07-523, a CD4 binding site-specific mAb that is undergoing clinical trials, had equivalent breadth and somewhat greater potency. The neutralization patterns observed with the CD4/7B2 chimeras most closely resembled those of the two mAbs directed against the membrane proximal external region of gp41: 10E8 and 4E10.

### 3.5. Ab-Dependent Phagocytosis

**Figure 6:**
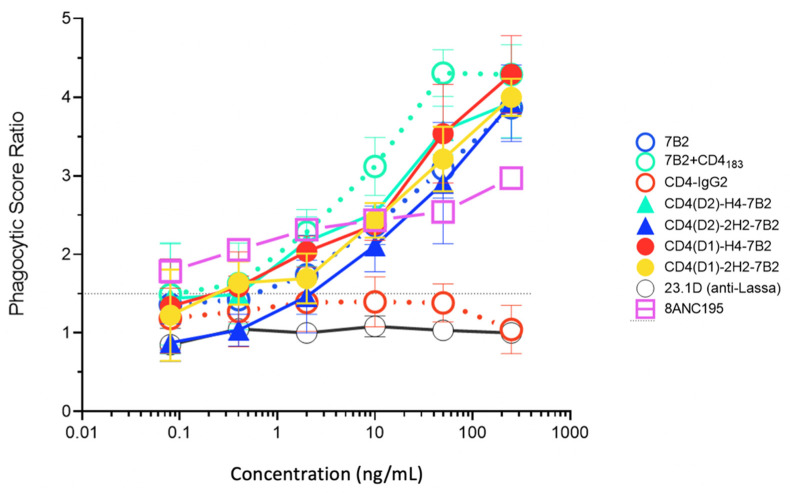
Antibody-dependent phagocytosis with panel mAbs and Ig-adhesins. We quantified the ingestion of gp140-coated fluorescent beads by THP-1 cells. Beads were opsonized with the indicated Ab. The novel Ig-adhesins are represented as solid lines and symbols, while the controls are dotted lines and open symbols. For each concentration, the phagocytic score and SEM are shown. When no error bars are visible, they are smaller than the symbol. The horizontal line indicates the cutoff for significance. The data shown are representative of three separate experiments.

Fc-mediated effector functions are critical for the ability of even neutralizing Abs to function most effectively in vivo [16,37,38,39,40,41,42,43,44,45]. Other Fc-mediated antiviral effects of Ab include phagocytosis of virions [67], lysis of HIV-infected cells by ADCC [44,45], or a combination of these activities [70]. We tested our panel of CD4/7B2 chimeras and controls for their ability to opsonize fluorescent microspheres coated with oligomeric gp140 for ingestion by THP-1 cells (Figure 6). Among the parental mAbs, 7B2 was consistently better than CD4-IgG2 at mediating ADP. We cannot say with certainty if this is a function of epitope specificity (gp41 better than gp120), or the isotype of the Ab (IgG1 vs. IgG2). When CD4_183_ was added to 7B2, greater phagocytosis is observed. The four immunoadhesins all mediated ADP, approximately as well as 7B2 alone, but not as well 7B2 + CD4_183_.

## 4. Discussion

It is our goal to develop immunoconjugates that could be used to eradicate persistent reservoirs of HIV infection. In comparing the use of different mAbs to deliver cytotoxic agents, including many broadly neutralizing and potent mAbs, we have consistently identified the gp41 external heptad repeat (HR)/disulfide-loop region as the most effective target of cytotoxic immunoconjugates, but only when used in association with sCD4 [46,47,48,49,68]. For this reason, we sought to produce a single construct that fulfilled the role of both anti-gp41 mAb and sCD4. Using design principles derived from our prior studies of gp120/gp41-bispecific double variable domain Abs [24], we made similar constructs adding the NH_2_-terminal domains of CD4 to the amino terminus of either the H chain, L chain, or both chains of the anti-gp41 mAb 7B2. Constructs in which CD4 was attached to the L chain were not secreted in sufficient quantity to analyze. We then compared these four immunoadhesins (two different CD4 constructs and two different linkers for each) to the parental proteins (7B2 and sCD4) individually and in combination, and to the well-established broadly neutralizing mAb 8ANC195, which binds determinants at the interface of gp120 and gp41. In general, the function of the chimeric immunoadhesins was intermediate between that of the parental components, with the exception of neutralization (Figure 5). We were surprised to find that our CD4/7B2 constructs had potent and very broad neutralizing activity. As for their ability to deliver cytotoxic immunoconjugates, the function for which they were designed, the chimeric constructs were not equal to a mixture of the two parentals.

MAb 7B2 neutralizes only weakly, if at all (Figure 5A), although recent studies suggest that mAbs to this region may have neutralizing potential when engaged by FcγRI [71]. It binds well to a highly conserved epitope. Beyond the additive neutralization of 7B2 and sCD4, other factors may play a role in the enhanced neutralization efficacy of the CD4/7B2 chimeras over the original parental components. Cross-linking of gp120 to gp41 by the bispecific immunoadhesin may be one such mechanism. The dissociation of gp120 from gp41 is a critical step in Env-mediated virus/cell fusion and infection. Natural mAbs and genetic constructs that bind to both gp120 and gp41 have been described, which neutralize HIV quite well [25,34,35], providing support for this hypothesis. However, other mAbs and constructs with demonstrated binding to both gp120 and gp41 neutralize poorly [24,33], suggesting other mechanisms are also involved.

We have found that combining two different gp160-binding domains on one molecule has differential effects on different functions of the chimeric adhesin: neutralization is markedly enhanced (Figure 5), whereas the ability to deliver a cytotoxic immunoconjugate is diminished (Figure 4). The discrepancy between neutralization and cytotoxicity is not unexpected, since cytotoxicity requires binding, internalization, intracellular routing, and processing of the cytotoxic conjugate [48,72,73]; whereas neutralization primarily occurs before the virus can enter the cell. In previous work, we showed that there is little correlation between the cytotoxic activity of different anti-gp160 mAbs and their ability to bind cell-surface Env or neutralize a virus secreted by those cells [46]. These results emphasize the notion that for anti-HIV Env Abs, and probably other antiviral mAbs, analysis of binding function and neutralization may not fully define their functional activity and therapeutic utility. This is consistent with findings that emphasize the role of Fc-mediated effector function in protective efficacy [16,37,38,39,40,41,42,43,44,45]. To enhance the antiviral utility of the CD4/7B2 chimeric adhesins described here, we engineered the Fc-associated glycans and studied the effects of these altered glycans on Fc-mediated effector functions.

## 5. Conclusions

We describe the production of engineered immunoadhesins that bind to both gp120 and gp41 of the HIV-envelope. The adhesin contains CD4 to bind gp120, and the anti-gp41 mAb 7B2. We tested these adhesins for binding, effector function, virus neutralization, and the ability to deliver cytotoxic immunoconjugates. In most assays, these adhesins functioned as well as either of the parental molecules. However, the adhesins showed broad and potent HIV-neutralizing activity exceeding that of either parent, even when 7B2 and CD4 were mixed together. These adhesins may be used therapeutically to treat active infection or to eliminate reservoirs of HIV-infected cells that persist despite antiviral therapy. This study also informs us about how Abs neutralize HIV, suggesting that by binding to both portions of the envelope, these Abs neutralize viruses by limiting the structural changes in the envelope that are necessary to cause infection.

## Figures and Tables

**Table 1:** Description of CD4-7B2 chimeric immunoadhesins.

Name	H Chain	L Chain	CD4	Linker
CD4(D1)-2H2-7B2	CD4(D1)-2H2-7B2	7B2k	CD4(D1.22)	(GGGGS)_2_-A(EAAK)_4_A-(GGGGS)_2_
CD4(D1)-H4-7B2	CD4(D1)-H4-7B2	7B2k	CD4(D1.22)	A(EAAK)_4_A
CD4(D2)-2H2-7B2	CD4(D2)-2H2-7B2	7B2k	CD4_183_	(GGGGS)_2_-A(EAAK)_4_A-(GGGGS)_2_
CD4(D2)-H4-7B2	CD4(D2)-H4-7B2	7B2k	CD4_183_	A(EAAK)_4_A

## Data Availability

All data are presented in the manuscript and Supplementary Table S1.

## Supplementary Materials

The following are available online at https://www.mdpi.com/article/10.3390/vaccines9070774/s1, Table S1: Neutralization: raw data and statistical analyses.
